# Doxylamine-pyridoxine for nausea and vomiting of pregnancy randomized placebo controlled trial: Prespecified analyses and reanalysis

**DOI:** 10.1371/journal.pone.0189978

**Published:** 2018-01-17

**Authors:** Navindra Persaud, Christopher Meaney, Khaled El-Emam, Rahim Moineddin, Kevin Thorpe

**Affiliations:** 1 Department of Family and Community Medicine and Li Ka Shing Knowledge Institute, St Michael’s Hospital, Toronto, Ontario, Canada; 2 Department of Family and Community Medicine, University of Toronto, Toronto, Ontario, Canada; 3 Department of Pediatrics, University of Ottawa, Children’s Hospital of Eastern Ontario Research Institute, Ottawa, Ontario, Canada; 4 Dalla Lana School of Public Health, University of Toronto, Toronto, Ontario, Canada; the University of Sydney, AUSTRALIA

## Abstract

**Background:**

Doxylamine-pyridoxine is recommended as a first line treatment for nausea and vomiting during pregnancy and it is commonly prescribed. We re-analysed the findings of a previously reported superiority trial of doxylamine-pyridoxine for the treatment of nausea and vomiting during pregnancy using the clinical study report obtained from Health Canada.

**Methods and findings:**

We re-analysed individual level data for a parallel arm randomized controlled trial that was conducted in six outpatient obstetrical practices in the United States. Pregnant women between 7 and 14 weeks of gestation with moderate nausea and vomiting of pregnancy symptoms. The active treatment was a tablet containing both doxylamine 10 mg and pyridoxine 10 mg taken between 2 and 4 times per day for 14 days depending on symptoms. The control was an identical placebo tablet taken using the same instructions. The primary outcome measure was improvement in nausea and vomiting of symptoms scores using the 13-point pregnancy unique quantification of emesis scale between baseline and 14 days using an ANCOVA. 140 participants were randomized into each group. Data for 131 active treatment participants and 125 control participants were analysed. On the final day of the trial, 101 active treatment participants and 86 control participants provided primary outcome measures.

There was greater improvement in symptoms scores with doxylamine-pyridoxine compared with placebo (0.73 points; 95% CI 0.21 to 1.25) when last observation carried forward imputation was used for missing data but the difference is not statistically significant using other approaches to missing data (e.g. 0.38; 95% CI -0.08 to 0.84 using complete data).

**Conclusions:**

There is a trend towards efficacy for nausea and vomiting symptoms with doxylamine-pyridoxine compared with placebo but the statistical significance of the difference depends on the method of handling missing data and the magnitude of the difference suggests that there is no clinically important benefit employing the prespecified minimal clinically important difference or “expected difference” of 3 points.

**Trial registration:**

Clinical Trial NCT00614445

## Introduction

Doxylamine-pyridoxine (Diclectin, Diclegis; formerly Bendectin and Debendox) is commonly prescribed for the treatment of nausea and vomiting of pregnancy.[1–3] One published randomized controlled trial (RCT) of doxylamine-pyridoxine versus placebo, DIC-301, has been relied on as evidence of efficacy.[4] This RCT led to approval of the combination product by the United States Food and Drug Administration (FDA),[5] is cited in support of a clinical practice guideline recommendation for doxylamine-pyridoxine as the first line (grade I-A) treatment for nausea and vomiting of pregnancy[6] and is included in a Cochrane Collaboration systematic review.[7] The results of this trial have been reported in several publications between 2010 and 2016 [4, 8, 9] and a summary of a United States FDA review is also publicly available.[5] All of these previous reports conclude that doxylamine-pyridoxine is efficacious. The previous reports include different descriptions of the methodology and the outcomes.

According to clinical trial reporting guidelines, trials should either be conducted according to a prespecified protocol or, if there is good reason for changing the protocol, the reasons for changes should be reported.[10] In order to prevent selective reporting or misreporting, prespecified methodologies must be compared with reported results.[11–15] Publishing re-analyses of abandoned or misreported studies is now a recognized way to ensure research findings are used properly to improve care.[16]

The primary objective of DIC-301 was to compare the efficacy of doxylamine-pyridoxine to placebo in the treatment of nausea and vomiting during pregnancy. We present previously unpublished information about the methods and results of DIC-301 from the clinical study report that was submitted to Health Canada. A reanalysis is justified because there are differences between the previous reports of this trial [4, 9], this trial was pivotal in leading to United States FDA approval [5] and this medication is commonly prescribed [1–3] consistent with its guideline recommended first-line use.[6]

## Methods

### Data sources for prespecified analyses and re-analysis

We re-analysed participant level data from the DIC-301 trial according to the RIAT initiative.[16] We contacted the original authors and the journal that published two prior reports of this trial. We used three sources of information about the prespecified analyses: publicly posted trial registration information, clinical study report documents obtained from Health Canada, and publicly posted review documents from the FDA. We describe the trial methodology based on these sources as well as the published articles about this trial.

We obtained individual participant level data from a .pdf file of the clinical study report from Health Canada. We converted the data tables in the obtained .pdf file into .doc and .xls files and then used the *R* statistical computing environment to clean and format the data.[17]

We compared findings with those reported in the only two articles where the stated purpose is to report the efficacy results of the trial.[4, 9]

For adverse events, we compared findings with those in the article about maternal safety results from the trial [8] and the information posted on the trial registration website.

### Design

This was a parallel, individual participant 1:1 randomized, superiority, multicenter, double-blind placebo controlled trial.

### Changes to protocol

The timing of primary outcome ascertainment, the analysis plan for the primary outcome, the secondary outcomes, and the sample size justification were apparently changed after the study was completed although it is unclear if changes to the analysis plan were made prior to initiating analysis. These changes are discussed in detail below and some are listed in Table A and Table B in S1 File.

### Participants and setting

Pregnant women aged 18 years or older with a gestational age between 7 and 14 weeks with nausea and vomiting or pregnancy symptoms (NVP) of at least 6 on the pregnancy unique quantification of emesis scale (PUQE score) despite conservative treatment were recruited from six university medical centres in the United States. Inclusion and exclusion criteria are listed in Box 1.

The six sites were the University of Texas Medical Branch in Galveston, the University of Texas Medical Branch Obstetrical Regional Maternal Clinic in Pasadena, University of Texas Medical Branch Regional Maternal & Child Health Program Clinic in Pearland, Magee Women’s Hospital in Pittsburgh, Washington Hospital Centre in Washington DC, and Georgetown Medical University in Washington DC.

**Box 1. Inclusion and exclusion criteria (clinical study report, page 24)**

**Inclusion criteria**

Subjects were eligible for study inclusion if they met all of the following inclusion criteria:

1. The subject had signed a written informed consent to participate in the study and had agreed to follow dosing instructions and complete all required study visits.
2. The subject was a pregnant female age equal to or greater than 18 years old.
3. The subject’s entry ultrasound indicated a viable pregnancy and confirmed gestational age of the fetus was 7–14 weeks at the anticipated time of the first dose of study medication or placebo. If an ultrasound was done within 4 weeks of the admission visit, and results could be obtained, an additional ultrasound was not necessary.
4. The subject was suffering from NVP and had a PUQE score ≥6.
5. The subject had not responded to conservative management consisting of dietary/lifestyle advice according to the 2004 ACOG Practice Bulletin.
6. The subject agreed, if on a multivitamin, to continue on their current dose of multivitamin for the duration of the trial.
7. The subject did not plan termination of the pregnancy.

**Exclusion criteria**

Subjects were excluded from study participation if they met any of the following exclusion criteria:

1. The investigator confirmed the subject’s nausea and vomiting was of etiology other than NVP.
2. The subject had gestational trophoblastic disease or multifetal gestation.
3. The subject had a condition for which antihistamines, in the opinion of the investigator, were contraindicated (epilepsy, alcoholism, glaucoma, chronic lung disease, urinary retention, heart block, etc.).
4. The subject had used antihistamines, anticholinergics, dopamine antagonists, serotonin antagonists, ginger, or anti-emetic therapy (including acupressure, acupuncture, homeopathic remedies, medical hypnosis, relief bands, etc.) to treat NVP in the previous 48 hours or planned to do so during the study.
5. The subject was using drugs that had anticholinergic activity (e.g., tricyclic antidepressants).
6. The subject was taking multivitamins containing more than 10 mg of vitamin B6, or planned to do so during the study.
7. The subject was taking supplementary vitamin B6 in addition to any multivitamin preparation, or planned to do so during the study.
8. The subject was currently drinking any amount of alcohol.
9. The subject had any condition that might have interfered with the conduct of the study.
10. The subject was likely to be unable to comply with study procedures because of inadequate cognitive skills.
11. The subject had received an investigational drug within 30 days before enrollment in this study or was scheduled to receive an investigational drug during the course of this study.

#### Patient involvement

Apparently there was no involvement of patients in the design of study. We did not involve patients in the re-analysis.

### Interventions

The study drug was either a combination tablet containing 10 mg of doxylamine and 10 mg of pyridoxine (vitamin B6) in a delayed-release formulation or a placebo.

Participants were instructed to take two tablets of the study drug on the first night of the study. If nausea and vomiting symptoms persisted (PUQE > 3) during the afternoon of day 2, participants were instructed to take an additional tablet on the morning of day 3. The need for an additional afternoon tablet was made during a clinic assessment on day 4 ± 1 (with a threshold of PUQE > 3). In summary, participants were instructed to take between 2 and 4 tablets daily according to their symptoms.

### Primary outcome and changes

According to the clinical study report, the “the primary efficacy endpoint was the change from baseline in PUQE score at Day 15 (± 1 day). Change from baseline was calculated as post-baseline score minus baseline score.”

Minor changes to the timing of primary outcome ascertainment were made and the quality of life score is also described as a primary outcome in a 2016 report (see Table C in S1 File).

### Secondary outcomes and changes

The secondary outcomes were: the three subscores of the PUQE, global assessment of wellbeing, number of tablets taken, time loss from household tasks and/or employment, total number of visits and phone calls to health care providers, rates of hyperemesis gravidarum and compliance with study medication.

The secondary outcomes were reported differently in two published articles (see Table D and Table E in S1 File).

### Sample size estimation

The sample size seems to be based on a clinically important difference of 3 on the 13-point PUQE score between groups, power of 90%, and an alpha of 0.001. The source cited is an RCT of ginger versus pyridoxine for the treatment of nausea and vomiting of pregnancy. Different justifications of the sample size are presented in different sources (see Table F in S1 File).

### Interim analyses

No interim analyses or stopping rules were described.

### Randomization: Sequence generation, type and allocation concealment mechanism

According to the clinical study report, “study drug was provided according to a computer-generated randomization code in blocks of a predetermined number of subjects. Block size was not disclosed to study center or study management personnel. If the subject was withdrawn from the study prior to Day 15 for any reason or in the event of an emergency, the site was to contact the medical monitor before breaking the blind. The randomization code was provided to the site by the interactive voice recording system. Subjects were assigned study medication sequentially, in the order of enrollment.”

### Blinding

The trial was described as “double-blind” and this presumably means that patient and clinicians were blinded to group assignment.

### Similarity of interventions

According to the clinical study report, “the placebo tablets were of identical in size, shape, taste, and color” as the doxylamine-pyridoxine tablets and only the lot number differed.

### Statistical re-analysis: Data extraction

We obtained the clinical study reports including individual participant level data from Health Canada under a confidentiality agreement. We extracted the individual level participant data that was provided by Health Canada in the form of .pdf file with multiple data tables in two ways: (1) by manually copying and pasting relevant data elements into a spreadsheet and (2) using software to automatically extract data from the tables. We compared the results of both extraction techniques and resolved discrepancies by referring to the original .pdf file.

### Statistical re-analysis: Reproducing pre-specified analyses

We use the term "prespecified" to refer to analyses specified before or around the start of the inclusion of the first participant which occurred on 7 February 2008 according to the clinical study report (page 1). The prespecified analyses are generally those described in the statistical analysis plan dated 29 May 2008.

### Statistical re-analysis of primary outcome: Reproducing pre-specified analyses

As prespecified in the original protocol, we employed an ANCOVA with the change from baseline to day 15 ± 1 as the response variable and the baseline symptom score as the covariate.

For missing data, a last-observation-carried forward approach was employed as pre-specified in the original protocol. According to the statistical plan, a sensitivity analysis employing only participants with complete data was also prespecified in order “to demonstrate that study conclusions are invariant to assumptions, the particular model, and methods of handling missing data” according to the statistical analysis plan. Later statistical analysis plans describe performing this sensitivity analysis on per protocol participants (who have a day 15 ± 1 symptom scores and who took between 80% and 120% of study drugs). We present both sensitivity analyses. The prespecified primary outcome analysis plans in different sources are summarized in Table G in S1 File.

Methods for post-hoc analyses related to the handling of missing data are presented in the supporting information section.

### Secondary outcomes

We present the secondary outcome analyses from the clinical study report. According to the clinical study report, only available data were used for some secondary outcomes but for the following secondary outcomes missing data were imputed using the “period mean”: time loss from household tasks, time loss from employment, number of visits and number of phone calls to health care providers.

### Statistical re-analysis of adverse events

As prespecified we present all adverse effects that occurred between day 1 and day 15. We also report all adverse events recorded. As prespecified, we present the results of a Pearson’s chi-squared test to compare adverse effects between treatment groups.

### Data sharing

The underlying data may be available upon request to Health Canada. Our requests under the Access to Information Act and the Protecting Canadians from Unsafe Drugs Act (Vanessa’s Law) to publicly post the underlying data were denied by Health Canada (see S1 Letter).[18] In order to obtain the information, we had to sign a confidentiality agreement with Health Canada that prevents us from sharing the clinical study report including the individual participant level data.

We also requested the clinical study reports and underlying data from the European Medicines Agency who responded that they had no such information to provide. We made a request to the United States FDA but were told that we may not receive a response for several years. We requested the individual participant level data from the trial sponsor, Duchesnay Inc., but received no response.

## Results

**Fig 1:**
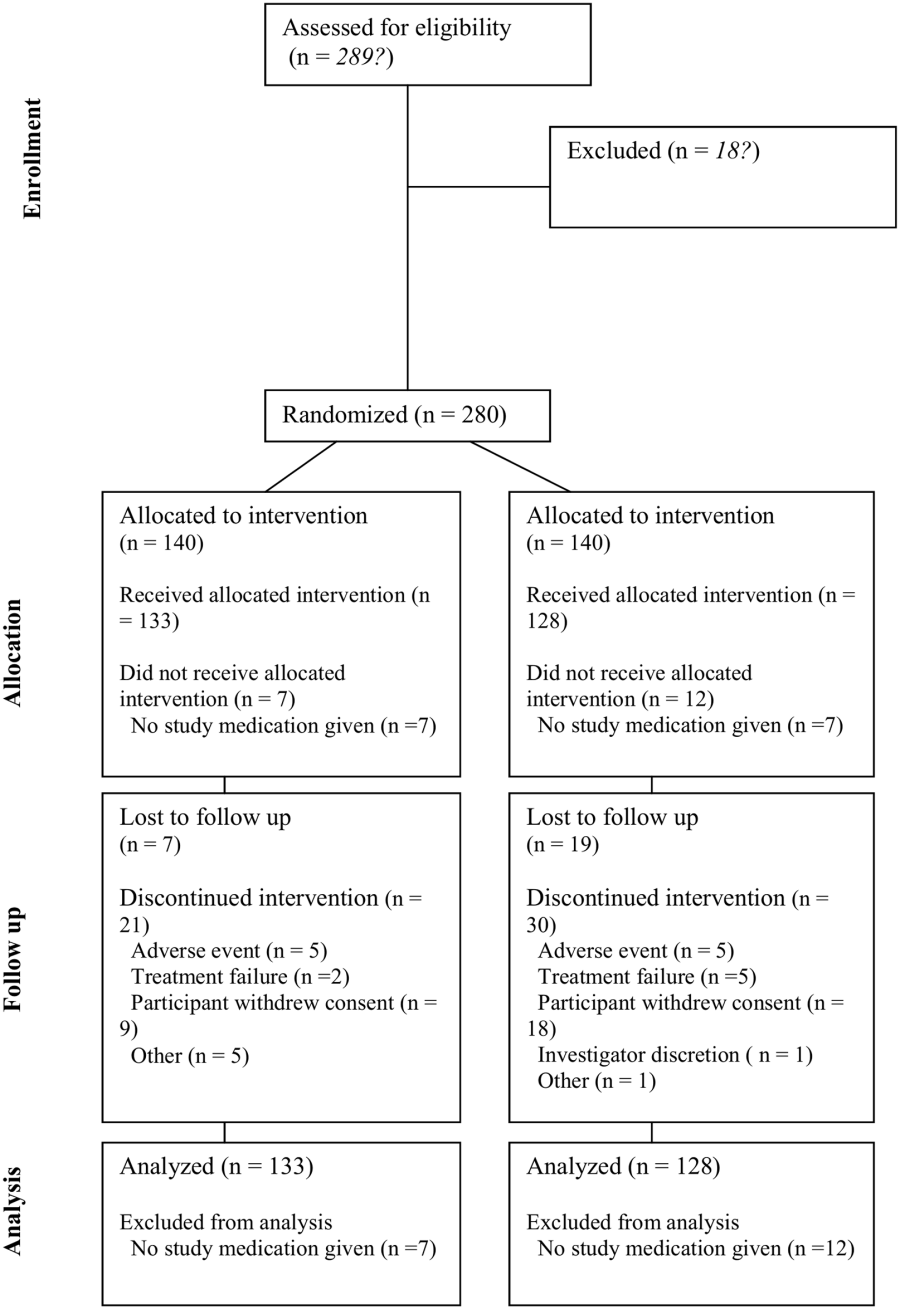
CONSORT flow diagram for “ITT-Efficacy” group based on clinical study report (page 6687).

Fig 1 summarizes the allocations and discontinuations for the two treatment groups. Additional information about the disposition of participants in the clinical study report showed that more participants allocated to the doxylamine-pyridoxine group were deemed to have completed the study: 112 (80%) versus 91 (65%) for the placebo group (p = 0.007, chi-squared 7.2) (Table H in S1 File). Table 1 summarizes the baseline characteristics of participants in the two treatment groups.

**Table 1:** Baseline characteristics of participants.

	Doxylamine-pyridoxine (n = 131)	Placebo (n = 128)
**Maternal age**	25.9 (SD = 5.95)	25.0 (SD = 5.64)
**Race: Asian**	2 (1.5%)	1 (0.8%)
**Race: African American**	49 (37.4%)	49 (38.5%)
**Race: White**	80 (61.1%)	75 (59.1%)
**Race: Other**	0	2 (1.6%)
**BMI**	28.8 (SD = 7.6)	29.8 (SD = 11.1)
**Start of NVP, gestational age, weeks**	5.47 (SD = 1.81)	5.34 (SD = 1.77)
**Enrollment, gestational age, weeks**	9.29 (SD = 1.96)	9.31 (SD = 1.83)
PUQE score at enrollment*	8.95 (SD = 2.11)	8.77 (SD = 2.10)
Global wellbeing at enrollment**	5.03 (SD = 2.32)	5.45 (SD = 2.19)

*The Pregnancy Unique Quantification of Emesis (PUQE) is a 13-point score ranging from 3 (no symptoms) to 15.

**The global wellbeing scale is an 11-point score ranging from 0 to 10.

### Primary outcome

**Fig 2:**
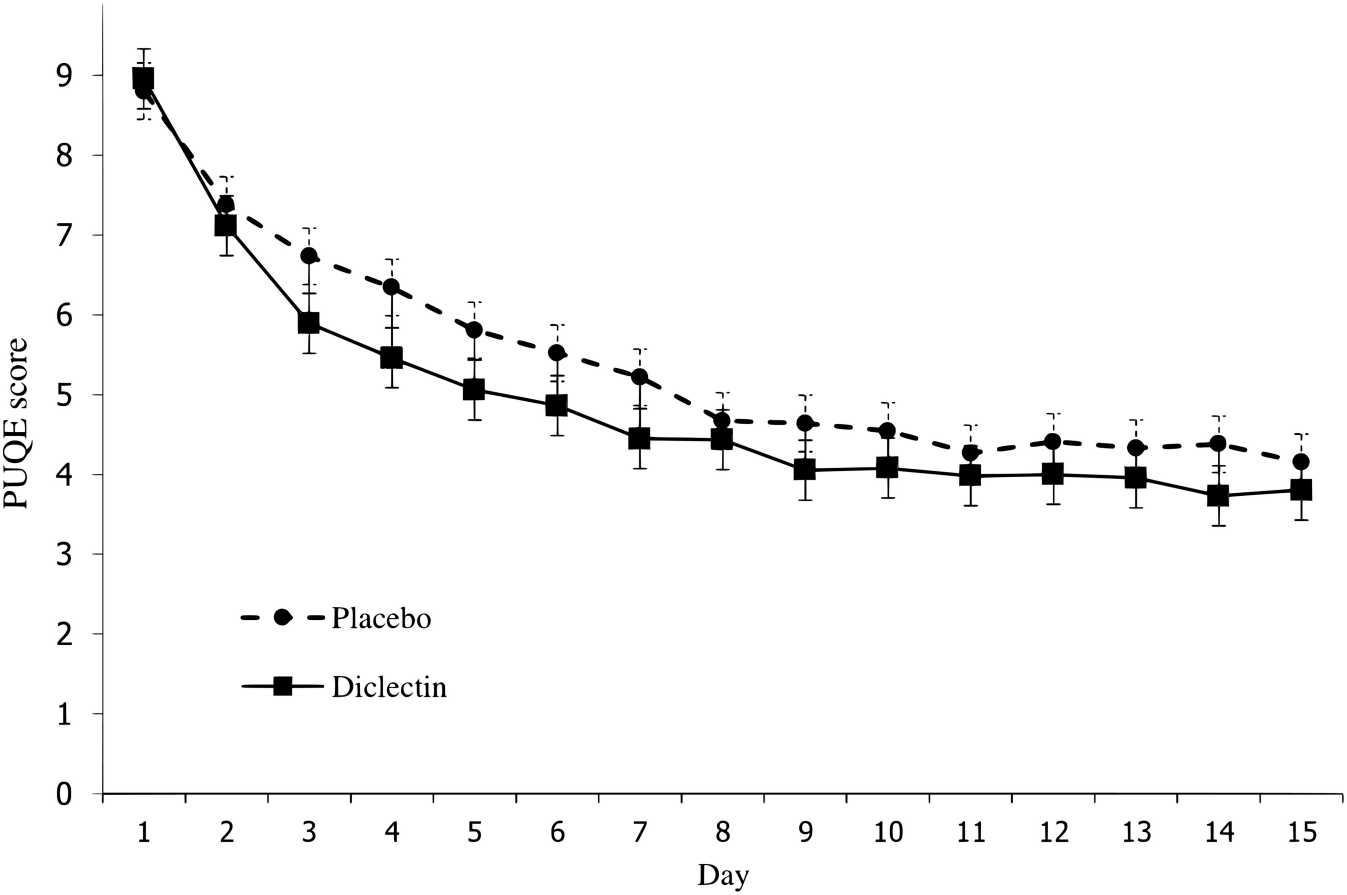
Mean symptom scores on each study day using available cases.

**Fig 3:**
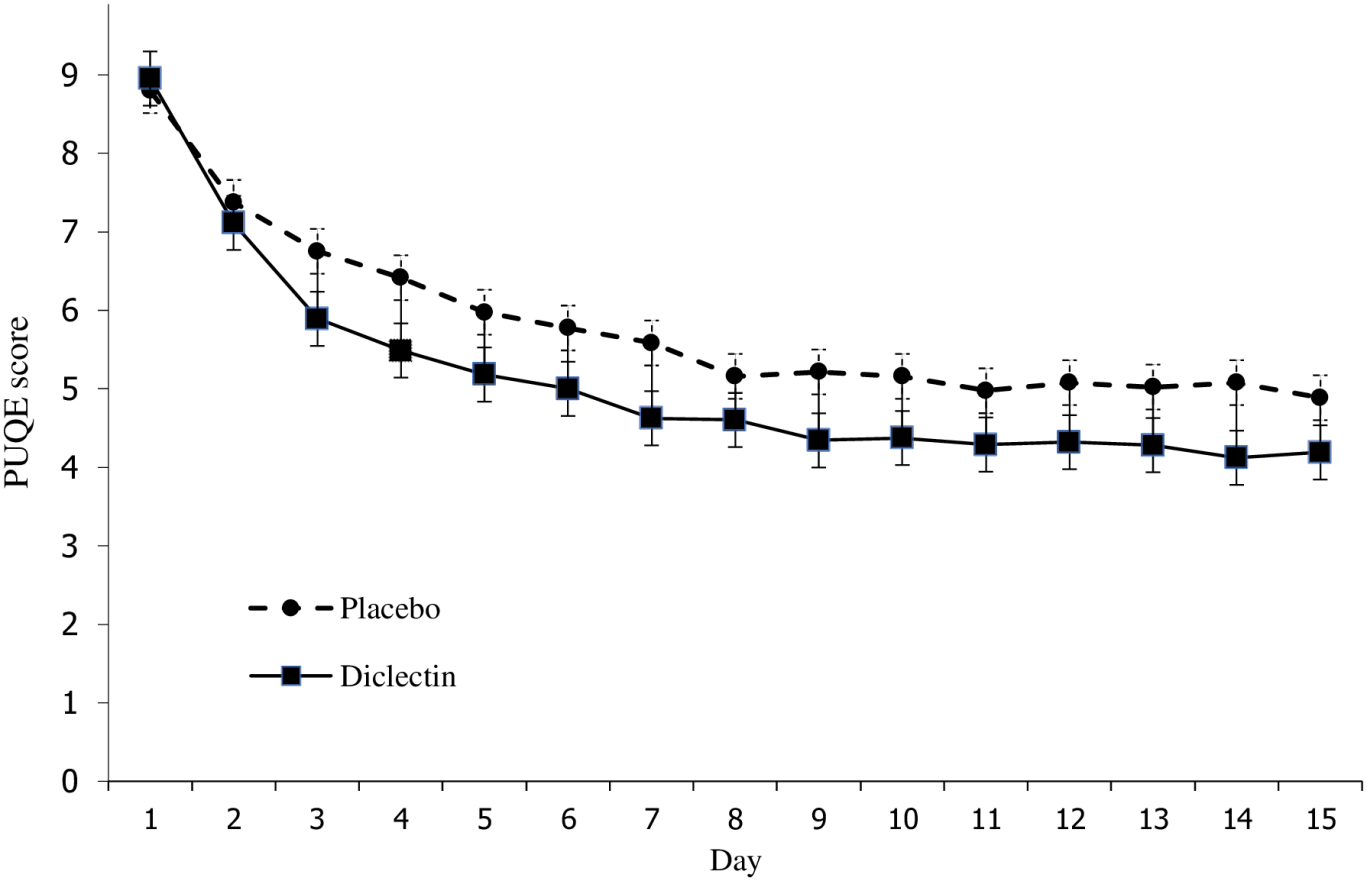
Mean symptom scores on each study day using last observation carried forward imputation.

Doxylamine-pyridoxine use led to a larger reduction in symptoms compared with placebo in the prespecified imputation using last observation carried forward analysis (LOCF) but no significant difference using the prespecified complete data sensitivity analysis (Table 2) (Figs 2 and 3). Fig 2 shows symptoms scores in each group during the trial. On the last day, the mean symptom score in the doxylamine-pyridoxine group was 3.8 (SD = 1.4) and in the placebo group it was 4.2 (SD = 1.2).

**Table 2:** Results of different analyses of the primary outcome.

Model	Missing Data	Difference between groups in 13-point symptom scores	95% CI	P-value
ANCOVA*	last observation carried forward	-0.73	-1.25, -0.21	0.006
ANCOVA*	Include only “complete data”	-0.38	-0.84, 0.08	0.107
ANCOVA*	Include only “per protocol”	-0.53	-1.02, -0.05	0.032
GEE difference-in-difference	Available Case	-0.45	-1.11, 0.21	0.186
GEE final symptom scores	Available Case	-0.31	-0.78, 0.16	0.203
LMM difference-in-difference	Available Case	-0.54	-1.12, 0.05	0.071
LMM final symptom scores	Available Case	-0.38	-0.94, 0.17	0.175

*Prespecified. ANCOVA = analysis of covariance; GEE = generalized estimating equation; LMM = linear mixed model

The results of the post-hoc analyses of different approaches to dealing with missing data are shown in Table 2.

### Secondary outcomes

Table 3 (left data column) summarizes the secondary outcome analyses. The results in the clinical study report showed that there were statistically significant differences based on a p = 0.05 threshold for global well-being but not for the other ten secondary outcomes. The 2010 article included global well-being and time lost from employment as well as two other outcomes that were not prespecified and did not include the other prespecified secondary outcomes. The 2016 letter included some of the outcomes that were not included in the 2010 article and indicated that some outcomes were not prespecified.

**Table 3:** Results of prespecified and reported analyses.

	Prespecified outcomes provided in clinical study report	Reported in 2010 *AJOG* article	Reported in 2016 *AJOG* article
**Primary outcome**			
PUQE total: LOCF imputation (doxylamine-pyridoxine vs placebo)*	-4.8 ± 2.7 vs -3.9 ± 2.6 (p = 0.006)	-4.8 ± 2.7 vs -3.9 ± 2.6 (p = 0.006)	(p = 0.006)
PUQE total: complete data*	-5.1 ± 2.5 vs -4.5 ± 2.5 (p = 0.18)	Not reported	Not reported
PUQE total: per protocol*	-5.3 ± 2.4 vs -4.6 ± 2.4 (p = 0.069)	Not reported	Not reported
Global well-being*	Not prespecified as primary outcome	Not clearly reported as primary outcome, see below	(p = 0.005)
**Secondary outcomes**			
PUQE component: nausea**	-2.6 ± 1.2 versus -2.5 ± 1.1 (p = 0.65)	Not reported	-2.6 ± 1.2 versus -2.5 ± 1.1 (p = 0.6)
PUQE component: vomiting**	-1.1 ± 1.2 vs -0.8 ± 1.2 (p = 0.084)	Not reported	-1.1 ± 0.3 vs -0.8 ± 1.2 (p = 0.08)
PUQE component: retching**	-1.5 ± 1.2 vs -1.3 ± 1.1 (p = 0.082)	Not reported	-1.5 ± 1.2 vs -1.3 ± 1.1 (p = 0.08)
Global well-being***	2.8 ± 2.8 vs 1.8 ± 2.2 (p = 0.005)	2.8 ± 2.8 vs 1.8 ± 2.2 (p = 0.005)	Not reported as secondary outcome, see above
Number of tablets taken	36.3 ± 13.3 vs 34.0 ± 15.1 (p = 0.14)	Not reported	36.6 ± 13.3 vs 34.0 ± 15.1 (p = 0.14)
Time lost from household tasks (hours)	6.09 ± 15.54 vs 5.51 ± 12.83 (p = 0.74)	Not reported	6.1 ± 15.5 vs 5.5 ± 12.8 (p = 0.73)
Time lost from employment (days)	0.92 ± 3.86 vs 2.37 ± 10.23 (p = 0.064)	0.92 ± 3.86 vs 2.37 ± 10.23 (p = 0.064)	Not reported
Visits to healthcare providers	0.1 ± 0.5 vs. 0.1 ± 0.4 (p = 0.89)	Not reported	0.1 ± 0.5 vs. 0.1 ± 0.4 (p = 0.88)
Telephone calls to healthcare providers	0.1 ± 0.4 vs. 0.1 ± 0.3 (p = 0.58)	Not reported	0.1 ± 0.4 vs. 0.1 ± 0.3 (p = 0.58)
Hyperemesis gravidarum	0 vs 0 (p-value not calculable)	Not reported	Not reported
Study drug compliance	68% vs 65% (P = 0.283)	Not reported	Not reported
Area under the curve change from baseline	Not prespecified	61.5 ± 36.9 vs 53.5 ± 37.5 (p 0.001)	Not reported (and not prespecified)
Compassionate use	Not prespecified	64 (48.9) vs 41 (32.8) (p = 0.009)	Not reported (and not prespecified)

* The Pregnancy Unique Quantification of Emesis (PUQE) is a 13-point score ranging from 3 (no symptoms) to 15.

**Each of the three Pregnancy Unique Quantification of Emesis (PUQE) subscores (for nausea, vomiting and retching) is a 5-points subscore ranging from 1 to 5.

***The global wellbeing scale is an 11-point score ranging from 0 to 10.

### Adverse effects

Table 4 summarizes the adverse events reported in the clinical study report. There were no important differences between groups.

**Table 4:** Most frequently reported treatment emergent adverse events and serious adverse events, as reported in clinical study report.

	doxylamine-pyridoxine (n = 133)	Placebo (n = 128)	p-value
**Headache**	17 (12.8%)	20 (15.6%)	0.51
**Somnolence**	19 (14.2%)	15 (11.7%)	0.58
**Fatigue**	9 (6.8%)	8 (6.3%)	0.86
**Dizziness**	8 (6.0%)	8 (6.3%)	0.94
**Back pain**	7 (5.3%)	4 (3.1%)	0.39
**Serious**			
**Bile duct stone**	0	1 (0.8%)	0.49
**Missed abortion**	1 (0.8%)	1 (0.8%)	1.0
**Spontaneous abortion**	2 (1.5%)	1 (0.8%)	0.49
**Fetal disorder**	0	1 (0.8%)	0.49
**Intrauterine death**	1 (0.8%)	0	1.0
**Premature rupture of membranes**	0	1 (0.8%)	0.49
**Withdrawals**			
**Withdrawals due to adverse events**	5 (3.8%)	5 (3.9%)	-

Most frequently reported treatment emergent adverse events are from page 6803 of clinical study report and serious adverse events are from page 6805 of clinical study report.

According to the clinical study report, there were 4 (3.0%) serious adverse events in the doxylamine-pyridoxine group and 5 (3.9%) in the placebo group. The same numbers are reported on the registration website. According to the article about maternal safety [8], there were 4 serious “treatment-emergent” adverse events in each group and separately 7 (5.3%) participants in the doxylamine-pyrodoxine group with at least one severe adverse event and 5 (3.9%) in the placebo group with at least one severe adverse event.

We reviewed the comments provided when women were explaining their global wellbeing score. We identified symptoms that were not recorded as adverse events although they could have been. These include several instances of somnolence and headache. There were apparently more of these events that were not reported as adverse events in the placebo group. These are summarized in Table H in S1 File (and for clarity they are not included in Table 4 above).

We also reviewed the overall study comments and found two adverse events that were not reported. There was one instance of dehydration resulting in an emergency department visit for intravenous rehydration on day 28,outside of the study period, in the doxylamine-pyridoxine group [page 5695 of clinical study report] that was recorded in the clinical study report as an instance of nausea and vomiting. A miscarriage on day 8 in the placebo group [page 5716 of clinical study report] was not recorded as an adverse event. These are summarized in Table J in S1 File.

## Discussion

### Principal findings and comparison with prior reports

The prespecified analyses showed a difference between treatment groups favoring doxylamine-pyridoxine that was not clinically important based on the prespecified “expected difference” or minimal clinically important difference and only statistically significant at the 0.05 alpha level using last observation carried forward imputation but not statistically significant using the prespecified sensitivity analysis involving only participants with complete data. Our post-hoc longitudinal analyses indicate a small treatment effect favoring doxylamine-pyridoxine that is neither statistically significant (at the 0.05 alpha level) nor clinically important.

In both groups, the symptom scores improve substantially over the two weeks of the trial and plateau after day 10 (Figs 2 and 3). The difference between groups also decreases after day 10. Both of these findings could be explained by the natural history of the condition, that is, by the resolution of symptoms irrespective of treatment. This natural resolution could account for the lack of clinical important difference between groups.

The clinically important difference for this trial was 3 points on the 13-point PUQE symptom score based on the “expected difference” in the original protocol. The trial of ginger versus pyridoxine for nausea and vomiting of pregnancy, that apparently was the basis for the sample size estimate, showed substantial improvements from baseline in both groups and no statistically significant difference between the ginger and pyridoxine groups on a 10-point visual analogue scale.[19] The sample size calculation for the trial of ginger versus pyridoxine was based on a clinically significant difference between groups of 25% (and an alpha of 0.05 and a beta of 0.20) which would roughly correspond to 3 units on the 13-point scale used here (25% of 13 = 3.25).

There are three studies that provide information about the clinical significance of different PUQE scores. A study of women calling a nausea and vomiting of pregnancy helpline categorized women having mild (4–6 points), moderate (7–12 points), or severe (13–15 points) symptoms based on the 12-hour version of the PUQE score. These categorizations were associated with discontinuation of multivitamins, healthcare costs and global wellbeing ratings.[20] The mean PUQE score for women who visited an emergency room or who were hospitalized (11 ± 3) was higher than for other women who called the helpline (9 ± 2.2). A similar study involving the same recruitment method and categorizations but employing the 24-hour version of the PUQE score found associations with the discontinuation of multivitamins and liquid intake.[21] In a validation study of the Norwegian version of the PUQE score, women hospitalized with hyperemesis gravidarum (median 13; 95% CI 11–14) had symptoms scores higher than women attending routine antenatal appointments (median 7; 95% CI 4–8).[22] The PUQE score was inversely correlated with nutritional intake.[22]

In summary, the pre-specified statistical analysis plan described an “expected difference” between doxylamine-pyridoxine and placebo of 3 points on the PUQE score whereas the largest estimate of the difference is 0.73 (95% CI: 0.21 to 1.25). The expected difference of 3 points is consistent with the clinically significant difference of 25% (3.25 on the 13-point PUQE scale) described in the ginger vs. pyridoxine study.[20] This is also consistent with the differences in PUQE scores associated with differences in outcomes such as emergency room visits among groups of women in the validation studies such as emergency room visits, 2 points in one study and 5 in another.[21, 23] Regardless of the analytical approach, the difference between groups was less than one point on the 13-point scale and there are no validation studies that indicate difference of this magnitude in this range of the scale (mild symptoms, one point above the minimum score) is clinically important.

The sample size calculation was apparently based on an alpha of 0.001 and an alpha of 0.05 is mentioned as the threshold for significance in the statistical analysis plan. Other values are provided in other sources (see Table F in S1 File). It is unclear why an alpha of 0.001 was used for the sample size calculation but an alpha of 0.05 was used to determine statistical significance. None of the analyses of the primary outcome indicate a p-value below 0.001 while some but not others are below 0.05. The large sample size was similar to that employed in an equivalence study of ginger and pyridoxine for the treatment of nausea and vomiting during pregnancy.[23] The results of this study might even be used to exclude the possibility that doxylamine-pyridoxine use provides a clinically important benefit over placebo. The large sample size in this trial provides informative results about the limited magnitude of effectiveness of doxylamine-pyridoxine. The maximum observed difference between drug treatment and placebo was 0.73 points (95% CI -1.25 to -0.21). The upper limit of this confidence interval, 1.25 points, suggests that a clinically important benefit is highly unlikely since 1.25 points is less than both the criterion originally specified in this trial (3 points) or the criterion used in the equivalence trial (2 points on a different 13-point scale).[24]

The efficacy outcomes were reported differently in the various publicly available documents related to this clinical trial (Table 3). The published articles report the results using the last observation carried forward imputation and not the sensitivity analyses, the global assessment of wellbeing is reported as a primary outcome when it was prespecified as a secondary outcome, some reported outcomes are described as secondary outcomes although they were not prespecified and some prespecified secondary outcomes were not reported.[4, 8, 9]

Given the limited magnitude of effectiveness observed in this trial, it is worth examining why the FDA approved doxylamine-pyridoxine following this trial. The summary of the FDA review described the outcomes as they were prespecified and described the prespecified sensitivity analyses involving the per protocol and complete data groups as showing no statistically significant difference between doxylamine-pyridoxine and placebo for the primary efficacy outcome.[5] The FDA statistical reviewer performed an additional sensitivity analysis (using an unspecified statistical test) that found a statistically significant difference of 0.49 on the PUQE scale using the per protocol group (p = 0.044) but not the complete data group (difference of 0.36, p = 0.15).[5] The trial sponsor responded to an FDA request by submitting the results of a “mixed model repeated measures analysis” with scores at all-time points (and apparently not just the end of study like the prespecified analysis) that found a statistically significant difference between groups (p = 0.0002). The FDA also considered the post-hoc analysis of the number of participants who requested compassionate use after the study which favored doxylamine-pyridoxine but the FDA apparently did not consider the prespecified secondary outcome of the number of tablets taken during the study where there was no difference between groups. The only information available about the “mixed model repeated measures analysis” is the results provided in the FDA review summary. Ultimately the FDA approved doxylamine-pyridoxine based on this trial. The FDA statistical review concluded that there was “some evidence that Diclegis [doxylamine-pyridoxine] was effective” and the medical review accepted the p-value of 0.006 and the difference between groups of 0.73 from the last carried forward imputation. The FDA summary review indicated a “small, but statistically significant improvement” and noted that “although the treatment effect is small, there are no other FDA-approved treatments for nausea and vomiting of pregnancy”.

Some secondary outcomes indicated a benefit of doxylamine-pyridoxine (e.g. time loss from employment, 0.92 ± 3.86 versus 2.37 ± 10.23 days, p = 0.064) while others indicated no difference between groups (e.g. time loss from household tasks). All PUQE subscores favored doxylamine-pyridoxine although the difference was not statistically significant; the difference between groups was smallest for nausea. The outcomes were reported differently in different publications (Table 3).[4, 9]

There was no important difference between groups in adverse outcomes even when previously unreported adverse events are considered. (This 15 day trial did not provide information about remote adverse events such as malformations which have been addressed through observational studies.)

Different information about the trial was obtained from different sources (Table 5). Trial reporting guidelines recommend that important changes to the methods be reported with reasons.[10] We were unable to discern the reasons for the differences between sources. No protocol deviations are mentioned in the 2010 report.[4] A table in the 2016 report of the trial describes some of the prespecified secondary outcomes as “previously unreported data”.[9]

**Table 5:** Selected differences between sources. Underlines indicate differences. Bolded text indicates consistency with the clinical study report. Italicized text indicates there is an apparent inconsistency with the clinical study report.

	Clinical study report	2010 AJOG article	2016 AJOG article	FDA Review
Sample size justification	“**The expected difference in PUQE scores between Diclectin and placebo is 3 (95 CI, 1–5)**."	“In recent studies on the effect of 500 mg ginger or 10 mg vitamin B6 on “nausea score” and on number of vomiting episodes, a sample size of 64 per group showed significant differences at power of 90% and alpha of .001.”	“One hundred and forty subjects per arm were to be enrolled to achieve 200 evaluable subjects for a power of 0.9 and beta of 0.01.”	“Per the application, **the expected difference in the PUQE scores between Diclegis and placebo is 3 (95% CI: 1–5)**…”
Number of individuals assessed for eligibility	**No mention found.**	*289*	**No mention found.**	**No mention found.**
Prespecified sensitivity analyses for prespecified primary outcome	**Both complete case and per protocol show trend towards efficacy but no statistically significant difference.**	No mention found	No mention found	**Both complete case and per protocol show trend towards efficacy but no statistically significant difference.**
Primary outcomes	**One**: Total symptom score change.	*One or two*: Total symptom score change. Global well-being also mentioned as primary outcome although not clear if primary or secondary.	*Two*: Total symptom score change and *global well-being*.	**One**: Total symptom score change.
Secondary outcomes	**(a) Three components constituting the PUQE;(b) Global assessment of well being;(c) Number of tablets taken;(d) Time loss from household tasks and or employment;(e) Total number of visits and phone calls to health care providers;(f) Rates of hyperemesis gravidarum;(g) Compliance with study medication**	Time lost from employment*“day-by-day area under thecurve for change in PUQE from baseline”*, *“number of women in each arm who continuedwith (blinded) compassionateuse of her medication (Diclectin or placebo)*.*”*	**Three components constituting the PUQENumber of tablets taken;Time loss from household tasks and or employment;Total number of visits and phone calls to health care providers;Rates of hyperemesis gravidarum;Compliance with study medication**	**Three individual components constituting the PUQE score, Global Assessment of Well-Being, Number of tablets taken,Time loss from household tasks and/or employment, Total number of visits and phone calls to healthcare providers, Rates of hyperemesis gravidarum**, *Relationship between levels of vitamin B6 (total and metabolites) anddoxylamine and PUQE score*
Conclusion	“Combination of the **primary, secondary, and exploratory endpoints indicate** clinically significant effects with theuse of Diclectin. The results of this study demonstrate Diclectin safety and **efficacy** in the treatment of nausea and vomiting of pregnancy when administered orally.”	“Diclectin delayed release formulation of doxylaminesuccinate and pyridoxine hydrochloride *is effective* and well tolerated in treating nausea and vomiting of pregnancy.”	“The prespecified primary endpoints and severalother non-prespecified parameters *support the effectiveness* of the pyridoxine-doxylamine delayed-release combinationover placebo.”	Statistical reviewer: “From a statistical perspective, the data submitted from the study DIC-301 provided some evidence that Diclegis was *effective* in the treatment of pregnant women with NVP. However, *clinical significance of such a small treatment effect and approvability decision is a clinical call*. Medical reviewer: “The data presented in the application for the single, placebo-controlled 15-day clinicaltrial supports the approval of Diclegis. A *statistically significant difference between Diclegis versus placebo was demonstrated* (p = 0.006). The analysis results confirmed atreatment improvement of -0.73 (95% CI, -1.25, -0.22) in pregnant women with NVP inthe ITT-E population via LOCF. Summary review: “…a new clinical trial that shows the Diclegis formulationprovides *a small*, *but statistically significant improvement* in nausea and vomiting ofpregnancy.”
Funding and potential conflicts of interest	“**Name of Sponsor/Company: Duchesnay Inc.”“Contract Research Organization: Premier Research Group Limited**”	“**The study was supported by Duchesnay Inc., Blainville, QC, Canada, and executed by Premier Research Group, Philadelphia, PA**.”“Dr Koren has served as aconsultant for Duchesnay Inc, Blainville, QC, Canada.”	“**The study was supported by Duchesnay Inc, Blainville, QC, Canada, and executed by Premier Research Group, Philadelphia, PA**”“The authors disclose the following: Dr Hankins: served on Scientific Advisory Board for Duchesnay USA; PI for FDA study that led to Diclectin approval in the United States; Dr Clark: Duchesnay Speaker Bureau; Dr Umans: consultant/Speaker for Duchesnay USA; Dr Koren: Has been a paid consultant by Duchesnay, which also supported some of his studies. Drs. Caritis, Miodovnik, and Mattison report no conflict of interest.”	Statistical and Medical review: “Applicant: **Duchesnay Inc**.”Summary review: “Applicant: *OptumInsight* for **Duchesnay Inc.**”

### Strengths and limitations

Our re-analysis was based on several documents including the clinical study report and appendices (see S1 Letter) that include the individual participant level data.

Our re-analysis employed the data submitted to regulatory bodies and was subject to all of the limitations of the original study including the substantial lost to follow up rate and the violations of the study protocol. In addition, we may have misinterpreted some aspects of the protocol or statistical analysis plan, our data extraction method could have introduced errors that were not detected (although several findings such as baseline characteristics were reproduced), and our re-analysis plan was developed only after we were aware of the trials reported findings. We were not allowed by Health Canada or the trial sponsor to make the underlying dataset publicly available so it will be challenging for others to reproduce the analysis.

There is no perfect method for dealing with missing data.[25, 26] Last observation carried forward imputation is known to be problematic in general because it assumes data are missing completely at random. In this trial last observation carried forward imputation increased (or worsened) the symptom scores in the control group relative to the active treatment group because there was more missing data in the control arm and symptom scores decreased or improved in both groups during the study). We employed the prespecified sensitivity analyses including subsets of participants that disregard data from excluded participants. According to the clinical study report the purpose of the sensitivity analysis using complete data was “to examine the impact of missing data and data imputation, and hence to demonstrate that study conclusions are invariant to assumptions, the particular model, and methods of handling missing data.” This prespecified approach to missing data is reasonable in certain settings and not uncommon.[27] The prespecified sensitivity analysis showed that the findings depended on how missing data were handled. A review of the trial by Health Canada in 2016 cited methodological issues including problems with the sample size calculation, reporting of reasons for dropouts, the last observation carried forward imputation, and the properties of the symptom scale in concluding that “the results of the study are not definitive and consequently the interpretation of the DIC-301 study is problematic.” [28]

We also employed two longitudinal models that utilize data from participants with incomplete measurements. These longitudinal models are not necessarily any better than the prespecified approach or other approaches such as multiple imputation and inverse weighting.[24, 25, 29] The longitudinal models allow for imbalanced longitudinal data under specific assumptions; the linear mixed model assumes data are missing at random and the linear generalized estimating equation model assumes data are missing completely at random.[30] Other methods for handling missing data with different strengths and limitations might yield different results. Some methods may indicate a statistically significant treatment effect while others may not. Even if additional analyses are performed we believe that inferences regarding treatment would be the same, since the difference between groups would still depend on the “assumptions, the particular model, and methods of handling missing data” based on the different findings presented in Table 2.

## Conclusions

This previously unpublished information about a trial of doxylamine-pyridoxine for the treatment of nausea and vomiting of pregnancy calls into question the conclusion of the original report that the medication is efficacious. For the primary outcome, none of the observed effect sizes exceeded the prespecified clinically important difference and inferences regarding the statistical significance of the treatment at a 5% alpha level are dependent on the handling of missing data. Clinical practice and guidelines should be updated. This reanalysis underscores the importance of public access to individual participant level data from clinical trials and the verification of their findings.

## Supporting information

S1 File Tables based on clinical study report and other sources. Table A shows the timeline of events in clinical trial DIC301. Table B shows the changes to protocol and explanations or changes provided in different sources. Table C shows the primary outcomes specified in different sources. Table D shows the description of outcomes in clinical study report analysis table headings. Table E shows the description of secondary outcomes in clinical study report analysis table headings. Table F shows the sample size justifications in different sources. Table G shows the primary outcome analysis plans in different sources. Table H shows the dispositions of participants based on clinical study report page 6687. Table I shows the events or symptoms recorded in comments but not reported as adverse events. Table J shows the events recorded in overall study comments but not reported as adverse events or secondary outcomes.
(DOC)

S1 Letter Decision letter from Health Canada refusing to publicly disclose the clinical study report. (PDF)
