# Supplementary material for: Doxylamine-pyridoxine for nausea and vomiting of pregnancy randomized placebo controlled trial: Prespecified analyses and reanalysis
Source: PLoS One. 2018 Jan 17;13(1):e0189978. doi: 10.1371/journal.pone.0189978 (PMC5771578; doi:10.1371/journal.pone.0189978)
Supplement: S1 File — Table A shows the timeline of events in clinical trial DIC301. Table B shows the changes to protocol and explanations or changes provided in different sources. Table C shows the primary outcomes specified in different sources. Table D shows the description of outcomes in clinical study report analysis table headings. Table E shows the description of secondary outcomes in clinical study report analysis table headings. Table F shows the sample size justifications in different sources. Table G shows the primary outcome analysis plans in different sources. Table H shows the dispositions of participants based on clinical study report page 6687. Table I shows the events or symptoms recorded in comments but not reported as adverse events. Table J shows the events recorded in overall study comments but not reported as adverse events or secondary outcomes. (DOC) [file pone.0189978.s001.doc]

**Doxylamine-pyridoxine for nausea and vomiting of pregnancy randomized placebo controlled trial: prespecified analyses and reanalysis: S1 Tables.** Tables based on the clinical study report and other sources.

**Table A**. Timeline of events in clinical trial DIC301.

| Date | Event | Source |
| --- | --- | --- |
| 18 April 2005 | Duchesnay submits a New Drug Application with the United States Food and Drug Administration for doxylamine-pyridoxine | FDA administrative and correspondence documents |
| 16 June 2005 | FDA refuses to file application | FDA administrative and correspondence documents |
| 7 February 2008 | First participant enrolled in DIC301 | Clinical study report page 2 |
| 12 February 2008 | First registration of primary outcome | Trial registration, clinicaltrials.gov |
| 29 May 2008 (signed 9 June 2008) | Statistical Analysis Plan version 1.0 | Clinical study report page 372 |
| 15 May 2009 (signed 18 June 2009) | Statistical Analysis Plan version 2.0 | Clinical study report page 440 |
| 16 June 2009 | Last participant completed study | Clinical study report page 2 |
| July 2009 | Final completion date (final data collection date for primary outcome measure) | Trial registration, clinicaltrials.gov |
| 5 August 2009 | Statistical Analysis Plan version 3.0 | Clinical study report page 512 |
| 14 December 2009 | Pre-New Drug Application between Duchesnay and Food and Drug Administration | FDA administrative and correspondence documents |
| 18 January 2010 | Clinical study report version 2.0 | Clinical study report page 1 |
| 12 February 2010 | American Journal of Obstetrics and Gynecology receives manuscript describing main trial results | AJOG 2010 paper |
| 21 July 2010 | American Journal of Obstetrics and Gynecology accepts manuscript describing main trial results | AJOG 2010 article |
| 8 June 2012 | Duchesnay re-submits New Drug Application | FDA approval letter |
| 8 April 2013 | FDA approves doxylamin-pyrodoxine | FDA approval letter |
| 25 January 2016 (received 14 January 2016) | American Journal of Obstetrics and Gynecology accepts for publication new manuscript describing trial results | AJOG 2016 article |

**Table B.** Changes to protocol and explanations or changes provided in different sources.

| Source | Primary outcome | Date |
| --- | --- | --- |
| Public registration | Primary outcome description and objective changed. Results added. | 2 September 2011 |
| FDA review document | Protocol Amendments for Study DIC-301:  The original protocol for Phase 3 Study DIC-301 was submitted to IND 72300 on December 21, 2006. The first subject enrollment in Study DIC-301 was on February 7,  2008. The last subjects completed Study DIC-301 on June 16, 2009. In total, 4 amendments were submitted to the original 2006 protocol: Amendment 1 dated March 27, 2007 reduced the number of study sites, increased the  PUQE eligibility requirement score (PUQE score > 4 was increased to PUQE score ≥ 6), reduced the requirement for post-baseline PUQE measurements (from twice daily, am  and pm, to once daily in the morning at approximately the same time each day), revised text to correct inconsistencies and accurately reflect revisions to the statistical portions  of the protocol, added drug accountability at each visit, improved schedule of events footnotes for clarity, readability, the 24 hour score, revised text to accurately reflect the current version of the Global Assessment of Well-Being used and the days on which it was performed, revised compassionate use to include AE collection within the first 4  weeks after the end of study, corrected procedural and statistical errors, and revised the schedule for blood sample collection to allow for greater flexibility. Amendment 2 dated June 7, 2007 defined the vitamin B6 metabolites to be evaluated, changed clinic evaluations from midday to morning, removed age restrictions as an inclusion criterion (changed from “pregnant female equal to or greater than 18 years old” to “patient is a pregnant female”), clarified treatments excluding subjects from study participation, clarified scheduling and content of PUQE evaluation and diary completion, added a serum chemistry analyte, and allowed for down-titration of study drug in the case of related AEs. Amendment 3 dated September 13, 2007 added back the minimum age criterion for study inclusion (“pregnant female equal to or greater than 18 years old”), clarified schedule of events, changed the Global Assessment of Well-Being scale, clarified days of diary recording, determined that subjects were required to have a clinic visit every 4 weeks during compassionate use, and clarified wording of the Global Assessment of  Well-Being questionnaire. Amendment 4 dated May 20, 2008 clarified dosing procedures, allowing for subjects to  take additional study drug if their symptoms of nausea and vomiting (PUQE score above 3) were not controlled by standard study drug administration (2 tablets), and  increased the total subject number from 260 to 280 to account for non-compliance and drop-out subjects.  The final Clinical Study Report for Study DIC-301 is dated January 18, 2010. | 26 February 2013 (page 49-50, FDA medical review) |
| Clinical study report | There were 4 amendments to the original protocol dated 21 Dec 2006 (Amendment 1 [dated  20 Mar 2007], Amendment 2 [dated 07 Jun 2007], Amendment 3 [dated 13 Sep 2007], and  Amendment 4 [dated 20 May 2008]).  Amendment 1 reduced the number of study sites, increased the PUQE eligibility requirement  score, reduced the requirement for post-baseline PUQE measurements, revised text to correct  inconsistencies and accurately reflect revisions to the statistical portions of the protocol, added  drug accountability at each visit, improved schedule of events footnotes for clarity, readability, and accuracy, added planned subject enrollment numbers, replaced the 12 hour PUQE score with  the 24 hour score, revised text to accurately reflect the current version of the Global Assessment  of Well-Being used and the days on which it was performed, revised compassionate use to  include AE collection within the first 4 weeks after the end of study, corrected procedural and  statistical errors, and revised the schedule for blood sample collection to allow for greater  flexibility.  Amendment 2 defined the vitamin B6 metabolites to be evaluated, changed clinic evaluations  from midday to morning, removed age restrictions as an inclusion criterion, clarified treatments  excluding subjects from study participation, clarified scheduling and content of PUQE evaluation  and diary completion, added a serum chemistry analyte, and allowed for down-titration of study  drug in the case of related AEs.  Amendment 3 added back the minimum age criterion for study inclusion, clarified schedule of  events, changed the Global Assessment of Well-Being scale, clarified days of diary recording,  determined that subjects were required to have a clinic visit every 4 weeks during compassionate  use, and clarified wording of the Global Assessment questionnaire.  Amendment 4 clarified dosing procedures, allowing for subjects to take additional study drug if  their symptoms of nausea and vomiting (PUQE score above 3) were not controlled by standard  study drug administration (2 tablets), and increased the total subject number from 260 to 280 to  account for non-compliance and drop-out subjects.  […]  After database lock, additional exploratory analyses were generated for the following:  Summarization of the relationship between change from baseline in PUQE score on Day 15 and average plasma levels of clinical visits for the ITT-E population (Table 14.4.6.1).  Summarization of the number of subjects per treatment group who requested to continue receiving study drug at the end of the 15 day trial (Table 14.4.7).” | Page 38-39 |
| 2010 AJOG article | No changes described. | Received 12 February 2010, Accepted 21 July 2010 |
| 2016 AJOG article | “In 2010 we published in the Journal a primary paper examining the effectiveness of the pyridoxine-doxylamine delayed release combination in treating symptoms of morning sickness (1). We were asked by readers to report on other end points that were listed online in the study plan (clinicaltrials.gov – NCT00614445), but were not reported in the primary paper. The objective of this letter is to report these additional data.” | Received 14 January 2016, Accepted 25 January 2016 |

**Table C.** Primary outcomes specified in different sources

| Source | Primary outcome | Date |
| --- | --- | --- |
| Public registration | "Measure: The change in the Pregnancy Unique Quantification of Emesis (PUQE) score from baseline between Diclectin® and placebo. Time Frame: Day 14 of treatment" | 12 February 2008 |
| Statistical analysis plan | “An alpha level of 0.10 (α = 0.10) will be assumed to assess the significance of interaction  effects when analyzing appropriate primary and secondary efficacy endpoints through analysis of  variance (ANOVA) or analysis of covariance (ANCOVA) models.”  "The primary efficacy endpoint will be the change from baseline in PUQE score at Day 15/ET.  Change from baseline will be calculated as post-baseline score minus baseline value.” | 29 May 2008 (page 377) |
| Statistical analysis plan, after data collection | “An alpha level of 0.10 (α = 0.10) will be assumed to assess the significance of interaction  effects when analyzing appropriate primary and secondary efficacy endpoints through analysis of  variance (ANOVA) or analysis of covariance (ANCOVA) models.”  “The primary efficacy endpoint will be the change from baseline in PUQE score at Day 15 (± 1 day). Change from baseline will be calculated as post-baseline score minus baseline  value. | 5 August 2009 (Page 517) |
| FDA review document | “PUQE scores based on the ITT-E subject data via the last-observation-carried-forward  (LOCF) method were evaluated using an analysis of covariance (ANCOVA) model  where change from baseline to Day 15 (± 1 day) was the response variable, the  baseline PUQE score was the covariate, and the treatment group and study center were  the fixed effects.” | 26 February 2013 (page 50, FDA medical review) |
| 2010 AJOG article | “The primary effectiveness endpoint consisted of change from baseline in the 2 domains of the PUQE score…The quality of life domain of  the PUQE score incorporates patients’  report of their present well-being from  zero (worst possible) to 10 (best possible)… PUQE scores, based on the complete data, were evaluated using an ANCOVA  model, with change from baseline to day 15 (± 1 day) as the response variable, baseline PUQE score was the covariate, and the treatment group and study center were the fixed effects.” | Received 12 February 2010, Accepted 21 July 2010 |
| 2016 AJOG article | “The active combination was superior to placebo in the pre-specified primary outcomes of change in the Pregnancy-Unique Quantification of Emesis (PUQE) score (p=0.006) and in the measurement of quality of life (p=0.005).” | Received 14 January 2016, Accepted 25 January 2016 |

**Table D .** Description of outcomes in clinical study report analysis table headings

| Outcome | Clinical study report table heading | Source |
| --- | --- | --- |
| PUQE total: imputation | Primary Efficacy Analysis: Change from Baseline to Day 15 (± 1 day) in PUQE Score for ITT-E Population | Table 14.4.1.1 (page 6784) |
| PUQE total: complete data | Sensitivity Analysis: Change from Baseline to Day 15 (± 1 day) in PUQE Score | Table 14.4.1.2 (page 6785) |
| PUQE total: per protocol | Sensitivity Analysis: Change from Baseline to Day 15 (± 1 day) in PUQE Score | Table 14.4.1.3 (page 6786) |
| PUQE component: nausea | Secondary Efficacy Analysis: Change from Baseline to Day 15 (± 1 day) in PUQE Score Components: Hours of Nausea for ITT-E  Population | Table 14.4.2.1 (page 6787) |
| PUQE component: vomiting | Secondary Efficacy Analysis: Change from Baseline to Day 15 (± 1 day) in PUQE Score Components: Number of Times Vomited for  ITT-E Population | Table 14.4.2.2 (page 6788) |
| PUQE component: retching | Secondary Efficacy Analysis: Change from Baseline to Day 15 (± 1 day) in PUQE Score Components: Number of Times Retching  for ITT-E Population | Table 14.4.2.3 (page 6789) |
| Global well-being | Secondary Efficacy Analysis: Change from Baseline to Day 15 (± 1 day) in Global Assessment of Well-being for ITT-E Population | Table 14.4.3 (page 6790) |
| Number of tablets taken | Secondary Efficacy Analysis: Number of Tablets Taken, Time Loss, and Visit/Call to Health Provider for ITT-E Population | Table 14.4.4 (page 6791) |
| Time lost from household tasks | Secondary Efficacy Analysis: Number of Tablets Taken, Time Loss, and Visit/Call to Health Provider for ITT-E Population | Table 14.4.4 (page 6791) |
| Time lost from employment | Secondary Efficacy Analysis: Number of Tablets Taken, Time Loss, and Visit/Call to Health Provider for ITT-E Population | Table 14.4.4 (page 6791) |
| Visits to healthcare providers | Secondary Efficacy Analysis: Number of Tablets Taken, Time Loss, and Visit/Call to Health Provider for ITT-E Population | Table 14.4.4 (page 6791) |
| Telephone calls to healthcare providers | Secondary Efficacy Analysis: Number of Tablets Taken, Time Loss, and Visit/Call to Health Provider for ITT-E Population | Table 14.4.4 (page 6791) |
| Hyperemesis gravidum | Secondary Efficacy Analysis: Rates of Hyperemesis Gravidarum and Study Drug Compliance for ITT-E Population | Table 14.4.5 (page 6792) |
| Study drug compliance | *Not present in appendix* | *Not present in appendix* |
| Area under the curve total PUQE | *Not present in appendix* | *Not present in appendix* |
| Compassionate use | Exploratory Efficacy Analysis: Compassionate Use of Study Drug for ITT-E Population | Table 14.4.7 (page 6795) |

**Table E.** Description of secondary outcomes in clinical study report analysis table headings

| Source | Secondary outcomes | Date |
| --- | --- | --- |
| Public registration | No secondary outcomes described. | 12 February 2008 |
| Statistical analysis plan | “The secondary efficacy endpoints include:  (a) Three components constituting the PUQE;  (b) Global assessment of well being;  (c) Number of tablets taken;  (d) Time loss from household tasks and or employment;  (e) Total number of visits and phone calls to health care providers;  (f) Rates of hyperemesis gravidarum;  (g) Compliance with study medication (0 = less than 28 tablets, 1 = 28 tablets, 2 = more than  28 tablets).” | 29 May 2008 (page 378) |
| Statistical analysis plan, after data collection | “The secondary efficacy endpoints include:  (a) Three components constituting the PUQE;  (b) Global assessment of well being;  (c) Number of tablets taken;  (d) Time loss from household tasks and/or employment;  (e) Total number of visits and phone calls to health care providers;  (f) Rates of hyperemesis gravidarum;  (g) Compliance with study medication (0 = less than 28 tablets, 1 = 28 tablets, 2 = more than  28 tablets).” | 5 August 2009 (Page 519) |
| FDA review document | “The secondary efficacy endpoints in Study DIC-301 included:  ● the three individual components constituting the PUQE (hours of nausea, number of  times vomiting, and number of times retching),  ● Global Assessment of Well-Being,  ● number of tablets taken,  ● time loss from household tasks and/or employment,  ● total number of visits and phone calls to healthcare providers,  ● rates of hyperemesis gravidarum, and  ● compliance with study medication (0 = less than 28 tablets, 1 = 28 tablets, 2 = more  than 28 tablets).” | 26 February 2013 (page 61, FDA medical review) |
| 2010 AJOG article | “Secondary effectiveness criteria included the day-by-day area under the curve for change in PUQE from baseline, time loss from employment, and the number of women in each arm who continued with (blinded) compassionate use of her medication (Diclectin or placebo). We also recorded the number of patients in each group who reported concurrent use of alternate therapy for NVP.” | Received 12 February 2010, Accepted 21 July 2010 |
| 2016 AJOG article | “No. Of tablets taken, Time loss from household tasks (hr), No. visits to MD, No. of phone calls,  PUQE nausea change from baseline, PUQE vomiting change from baseline, PUQE retching change from baseline” | Received 14 January 2016, Accepted 25 January 2016 |

**Table F.** Sample size justifications in different sources.

| Source | Sample size justification | Date (and source) |
| --- | --- | --- |
| Public registration | “No text entered” | *Not applicable* |
| Statistical analysis plan | "In recent studies on the effect of 500 mg of ginger or 10 mg of vitamin B6 on “nausea score” and on number of vomiting episodes, a large effect size (Cohen d of 0.7-1) was measured, allowing a sample size of 64 per group to show significant differences at power of 90% and p-value of 0.001. The expected difference in PUQE scores between Diclectin® and placebo is 3 (95 CI, 1-5). Therefore, for this study, 280 patients (140 patients per treatment group) will be enrolled to achieve 200 evaluable patients. An estimated dropout rate of 25% and a non-compliance rate of approximately 5% are expected. This sample size is at least 4-fold larger than needed to show the intended clinical effect." | 29 May 2008 (page 376) |
| Statistical analysis plan, after data collection | “In recent studies on the effect of 500 mg of ginger or 10 mg of vitamin B6 on “nausea score” and on number of vomiting episodes, a large effect size (Cohen d of 0.7-1) was measured, allowing a sample size of 64 per group to show significant differences at power of 90% and p-value of 0.001. The expected difference in PUQE scores between Diclectin® and placebo is 3 (95% CI, 1-5). Therefore, for this study, 280 patients (140 patients per treatment group) will be enrolled to achieve 200 evaluable patients. An estimated dropout rate of 25% and a non-compliance rate of approximately 5% are expected. This sample size is at least 4-fold larger than needed to show the intended clinical effect.” | 5 August 2009 (Page 517) |
| FDA review document | “Per the application, the expected difference in the PUQE scores between Diclegis and placebo is 3 (95% CI: 1- 5); therefore, for this study, 280 subjects (140 subjects per treatment group) were to be enrolled to achieve 200 evaluable subjects. An estimated dropout rate of 25% and a non-compliance rate of approximately 5% were expected. This sample size was at least 4-fold larger than needed to show the intended clinical effect.” | 26 February 2013 (page 48, FDA medical review) |
| 2010 AJOG article | “In recent studies on the effect of 500 mg ginger or 10 mg vitamin B6 on “nausea score” and on number of vomiting episodes, a sample size of 64 per group showed significant differences at power of 90% and alpha of .001. Therefore, for this study, 280 patients (140 patients per treatment group) were enrolled to achieve 200 evaluable patients." | Received 12 February 2010, Accepted 21 July 2010 |
| 2016 AJOG article | “One hundred and forty subjects per arm were to be enrolled to achieve 200 evaluable subjects for a power of 0.9 and beta of 0.01. An estimated dropout rate of 25% and a noncompliance rate of approximately 5% were expected.” | Received 14 January 2016, Accepted 25 January 2016 |

**Table G.** Primary outcome analysis plans in different sources.

| Source | Primary outcome analysis plan | Date (and source) |
| --- | --- | --- |
| Registration | *No details provided* | *Not applicable* |
| Statistical analysis plan | “Two analysis populations, consistent with the protocol, are defined as follows.  (1) Intent-to-Treat efficacy (ITT-E) population: Any subject who took at least one dose of study medication and has at least one post-baseline PUQE measurement.  (2) Intent-to-Treat safety (ITT-S) population: Any subject who took at least one dose of study medication during the study.  For the purpose of determining the ITT-E status of the subject, a “study protocol violation” is defined as  any subject or investigator activity that could possibly interfere with the therapeutic administration of the treatment or the precise evaluation of treatment efficacy; subjects in this situation will be included in the ITT-S analyses, but excluded from the ITT-E analyses. However, subjects with any deviation from the protocol that would not interfere with the effect of, or the accurate assessment of, the assigned study  treatment may be included in both the ITT-E and ITT-S analyses.  The efficacy analyses will be conducted on ITT-E subject populations. Safety analyses will be conducted on the ITT-S subject population only.  […]  In the analyses of efficacy, for subjects who discontinue the study prematurely, a last-observation-carried-forward (LOCF) approach for the subsequent visits will be used for missing efficacy results in the ITT-E population for the Global Assessment of Well Being and PUQE score. For PUQE score, a subject has complete data if the subject has recorded PUQE scores for at least 7 of the 14 expected daily diaries from Day 2 to Day 14. The sensitivity analyses for PUQE Score will be conducted based on the complete data and imputed data to examine the impact of missing data and data imputation, and hence to demonstrate that study conclusions are invariant to assumptions, the particular model, and methods of handling missing data.” | 29 May 2008 (page 377) |
| Statistical analysis plan, after data collection | "Two analysis populations, consistent with the protocol, are defined as follows.  (1) Intent-to-Treat efficacy (ITT-E) population: Any subject who took at least one dose of study medication and has at least one post-baseline PUQE measurement.  (2) Intent-to-Treat safety (ITT-S) population: Any subject who took at least one dose of study medication during the study.  The efficacy analyses will be conducted on ITT-E subject populations. Safety analyses will be conducted on the ITT-S subject population only.  Two additional populations, subjects with complete data and per protocol subjects, will be used for  sensitivity purpose for primary efficacy analysis.  • A subject with complete data is defined as the subject who (a) has recorded baseline PUQE score,  (b) has recorded PUQE scores for at least 7 of the 14 expected daily diaries from the second day  of the subject’s maximal dose taken to Day 15 (± 1 day), and (c) absence of any major protocol  violations including the violation of entry criteria.  • A per protocol subject is defined as the subject who (a) has a valid baseline assessment, (b) has  recorded Day 15 (± 1 day) PUQE scores, (c) completed the study with between 80% - 120% of  prescribed study medication applications, and (d) absence of any major protocol violations  including the violation of entry criteria."  […]  The PUQE score based on 1) subjects with complete data via LOCF and 2) per protocol subjects  will be similarly performed separately for sensitivity purposes to examine the impact of missing  data and data imputation.” | 5 August 2009 (Page 519) |
| FDA review document | “The PUQE score based on 1) subjects with complete data via LOCF and 2) per protocol subjects were similarly performed separately for sensitivity purposes to examine the impact of missing data and data imputation.” | 26 February 2013 (page 48, FDA medical review) |
| 2010 AJOG article | “Analysis included all randomized patients who had received at least 1 dose of study medication.  […]  PUQE scores, based on the complete data, were evaluated using an ANCOVA model, with change from baseline to day 15 (± 1 day) as the response variable, baseline PUQE score was the covariate, and the treatment group and study center were the fixed effects.  Secondary analyses were used to compare the 2 treatment groups using ANCOVA where change from baseline to day 15 (_ 1 day) was the response variable, the baseline value was the covariate, and the treatment group and study center were the fixed effects.” | Received 12 February 2010, Accepted 21 July 2010 |
| 2016 AJOG article | Not described. | Received 14 January 2016, Accepted 25 January 2016 |

**Table H.** Dispositions of participants based on clinical study report page 6687.

|  | Doxylamine-pyrodoxine | placebo |
| --- | --- | --- |
| Enrolled | 140 | 140 |
| randomized | 140 | 140 |
| “Subjects Included in ITT-S Analysis” | 133 | 128 |
| “Subjects Included in ITT-E Analysis” | 131 | 125 |
| “Subjects Completed Study” | 112 | 91 |
| “Subjects Discontinued Study” | 28 | 49 |
| “Reason Discontinued:” |  |  |
| "Adverse Event (not including death)" | 5 | 5 |
| “Protocol Deviation” | 0 | 0 |
| “Subject Withdrew Consent” | 9 | 18 |
| “Investigator Discretion” | 0 | 1 |
| “Treatment Failure” | 2 | 5 |
| Subject Unblinding | 0 | 0 |
| Lost To Follow-up | 7 | 19 |
| Subject Death | 0 | 0 |
| Other | 5 | 1 |

**Table I.** Events or symptoms recorded in comments but not reported as adverse events

| Site | Group | Day | Page | Comment |
| --- | --- | --- | --- | --- |
| 10 | doxylamine-pyrodoxine | 1 | 4167 | MIGRAINES |
| 12 | doxylamine-pyrodoxine | 1 | 4177 | DIFFERENT FOODS. WAKING UP DIZZY LACK OF LATING |
| 12 | doxylamine-pyrodoxine | 14 | 4177 | SOME SLEEPINESS |
| 12 | doxylamine-pyrodoxine | 1 | 4178 | NAUSEA, VOMITING, HEADACHES, AND DIZZINESS |
| 12 | doxylamine-pyrodoxine | 1 | 4180 | HEADACHE, BACKACHE, NAUSEA |
| 12 | doxylamine-pyrodoxine | 1 | 4181 | I FEEL BROKEN DOWN, LETHARGIC, DUE TO THE NAUSEA |
| 20 | doxylamine-pyrodoxine | 1 | 4183 | TIRED - REAL HEAVY FEELING |
| 20 | doxylamine-pyrodoxine | 1 | 4184 | I GET IRRITATED EASILY NOW. |
| 20 | doxylamine-pyrodoxine | 1 | 4186 | JUST IRRITABLE |
| 20 | doxylamine-pyrodoxine | 1 | 4186 | I USUALLY SLEEP LESS |
| 20 | doxylamine-pyrodoxine | 8 | 4187 | JUST REALLY TIRED. |
| 20 | doxylamine-pyrodoxine | 8 | 4189 | BECAUSE I USUALLY SLEEPLESS. |
| 20 | doxylamine-pyrodoxine | 1 | 4190 | I USUALLY SLEEP LESS |
| 20 | doxylamine-pyrodoxine | 1 | 4190 | I NORMALLY DONT SLEEP DURING DAY. I AM NOW. |
| 20 | doxylamine-pyrodoxine | 1 | 4190 | I`M SICK AND TIRED. |
| 20 | doxylamine-pyrodoxine | 1 | 4190 | I CAN GET REAL EMOTIONAL |
| 30 | doxylamine-pyrodoxine | 14 | 4193 | MORNING SICKNESS AND HEADACHES |
| 31 | doxylamine-pyrodoxine | 1 | 4196 | VERY NAUSEATED, VOMITING & TIRED |
| 31 | doxylamine-pyrodoxine | 1 | 4198 | FATIGUE & NAUSEA |
| 31 | doxylamine-pyrodoxine | 8 | 4198 | TIRED, LESS NAUSEA IF SLEEPING. |
|  |  |  |  |  |
| 10 | placebo | 1 | 4200 | ALWAYS FEEL NAUSEATED, TIRED, SLEEPY, HUNGRY |
| 10 | placebo | 1 | 4200 | NO I WAS JUST VERY TIRED |
| 10 | placebo | 1 | 4201 | VOMITING, BORED, SLEEPY, HEADACHE |
| 10 | placebo | 1 | 4201 | I FEEL VERY TIRED I NORMALLY SLEEP 6 HOURS. I HAVE BEEN NAUSEATED AND VOMITING ALL WEEK AND ACID INDEGESTION |
| 10 | placebo | 1 | 4202 | FEEL TIRED, DIZZY AND NAUSEATED. |
| 10 | placebo | 1 | 4203 | RESTLESS |
| 10 | placebo | 1 | 4203 | INSOMNIA |
| 10 | placebo | 1 | 4203 | NAUSEA, HEADACHE & BACKACHES |
| 10 | placebo | 1 | 4204 | SLEEPY, NAUSEA, AND NO ENERGY |
| 11 | placebo | 1 | 4205 | MUCH NAUSEA AND VOMITING, TIRED |
| 11 | placebo | 8 | 4206 | BECAUSE NORMALLY I GO TO BED AT 9:00 PM, BUT NOW I CAN`T SLEEP UNTIL 2:00 IN THE MORNING. |
| 11 | placebo | 1 | 4206 | USUALLY SLEEP 6-8 HOURS |
| 11 | placebo | 1 | 4207 | I WAS SICK WITH: HEADACHE, NAUSEA, VOMITING |
| 11 | placebo | 8 | 4208 | NAUSEA, VOMITING, DIZZINESS AND HEADACHE. |
| 11 | placebo | 1 | 4208 | CAN`T SLEEP BECAUSE OF VOMITING & HEADACHE. |
| 12 | placebo | 1 | 4210 | I HAVE BEEN REALLY TIRED. |
| 12 | placebo | 1 | 4210 | I FEEL WITHOUT BREATH VERY SLEEPY AND NAUSEATED |
| 12 | placebo | 1 | 4211 | VOMIT, HEADACHE, DIZZINESS, FAINTINESS |
| 12 | placebo | 1 | 4212 | DUE TO NAUSEA AND SLEEPINESS. |
| 12 | placebo | 8 | 4212 | HEAD ACHE AND DIZZINESS |
| 12 | placebo | 1 | 4213 | NAUSEA, STOMACH DISCOMFORT, ABDOMINAL PAIN, HEADACHE, DIZZINESS (LATE NOTE) |
| 20 | placebo | 1 | 4215 | NAUSEATED - NO ENERGY -TIRED |
| 20 | placebo | 1 | 4216 | PREGNANCY - SHOCK - COPING AND GETTING BETTER WITH IT. |
| 20 | placebo | 1 | 4216 | BEING SICK AT MY STOMACH AND ALSO BEING TIRED. |
| 20 | placebo | 14 | 4217 | I JUST FEEL WONDERFUL EXCEPT FOR MY MIGRAINES |
| 20 | placebo | 8 | 4219 | BUMMED OUT |
| 20 | placebo | 1 | 4220 | VERY EMOTIONAL. USUALLY SLEEP MORE |
| 20 | placebo | 1 | 4220 | AM ALWAYS TIRED AND I CAN`T MOVE AROUND AS MUCH. |
| 20 | placebo | 1 | 4222 | SLEEPY BECAUSE I`M PREGNANT |
| 30 | placebo | 1 | 4225 | NAUSEA AND EXHAUSTION |
| 30 | placebo | 1 | 4226 | INCONSISTENT SLEEP AND EXTRA TIRED. |
| 30 | placebo | 1 | 4226 | I`M SLEEPY ALL THE TIME, NAUSEATED, AND DON`T HAVE A LOT OF ENERGY. |
| 30 | placebo | 1 | 4227 | I`M SO WEAK AND SICK. CAN`T SLEEP OR EAT NORMALLY. |

**Table J.** Events recorded in overall study comments but not reported as adverse events or secondary outcomes.

| Site | Group | Page | Comment |
| --- | --- | --- | --- |
| 20 | doxylamine-pyrodoxine | 5695 | PT IN EMERGENCY ROOM [day 28 of study] FOR IV HYDRATION PT D/C TO HOME. |
| 11 | placebo | 5716 | EARLY TERMINATION DUE TO SAE - SPONTANEOUS ABORTION |
