# Supplementary material for: Doxylamine-pyridoxine for nausea and vomiting of pregnancy randomized placebo controlled trial: Prespecified analyses and reanalysis
Source: PLoS One. 2018 Jan 17;13(1):e0189978. doi: 10.1371/journal.pone.0189978 (PMC5771578; doi:10.1371/journal.pone.0189978)
Supplement: S1 Letter — (PDF) [file pone.0189978.s002.PDF]

Health Canada
Santé Canada

Health Products and Food Branch
Direction générale des produits de santé et des aliments

250 Lanark Avenue
Graham Spry Building
A.L. 2007A
Ottawa, Ontario
K1A 0K9

Dr. Navindra Persaud
80 Bond Street
Toronto, Ont. M5B 1X2
nav.persaud@utoronto.ca

February 26, 2016

Dear Dr. Persaud,

I am responding to your request, dated January 29, 2016, "that Health Canada either (1) clarifies in writing that the patient level data in Appendix 16.2.10.1 of DIC301 may be publicly disclosed; or, failing that, (2) agrees to amend the confidentiality agreement so that the information in Appendix 16.2.10.1 can be publicly disclosed."

This information was disclosed to you in September 2015 under section 21.1(3)(c) of the *Food and Drugs Act*. Before it was disclosed to you, you agreed to terms that require all disclosed information to be kept confidential, subject to certain exclusions. These terms do not prevent publication of your findings, as long as the confidentiality of the disclosed information is maintained.

I am considering your request as a new request for disclosure of Appendix 16.2.10.1 under section 21.1(3)(c) without an obligation on you, the recipient, to keep the information confidential. In a letter dated February 12, 2016, you were informed of the basis of Health Canada's review and provided an opportunity to make further representations. You provided additional representations in support of your request in a letter dated February 17, 2016.

I have considered your representations in support of publicly releasing this data, including: benefits to patients, particularly pregnant women in Canada; informed consent of participants in the DIC301 clinical study; scientific rationale related to clinical trials in general, and to this study in particular; European Medicines Agency

publication of clinical trial data; and the importance of sharing individual participant level data.

I have also considered: expectations of the originator at the time the DIC301study was submitted to Health Canada; current Health Canada policy and practice with respect to public release of patient level data; and other options available to you under the current confidentiality agreement.

I am not prepared at this time to generally release the individual patient data contained in Appendix 16.2.10.1 to the DIC301 study to the public. I am not satisfied that the general disclosure of this information to the public is necessary to achieve the health promotion and protection purposes underlying this authority to disclose confidential business information. Nor am I satisfied that such a disclosure would sufficiently advance these purposes to warrant such a broad disclosure. Consequently, I wish to inform you that your request has been denied. Health Canada is prepared to consider requests from persons, such as members of a peer review panel, seeking access to the data in order to validate your analysis.

As you know, Health Canada will soon publish a Draft Guide that sets out in more detail the review process, principles and considerations for decisions regarding disclosure of Confidential Business Information under section 21.1(3)(c) of the FDA. This document will available in the coming weeks at: http://www.hc-sc.gc.ca/dhp-mps/prodpharma/applic-demande/guide-ld/index-eng.php. I urge you to provide your comments on the guidance document during the public consultation period.

I would like to thank you for the ongoing dialogue on this issue. I would also like to assure you that Health Canada is committed to further increasing public access to regulatory information. In recent years Health Canada has added significantly to the information it publishes on the Department's regulatory decisions, inspections and compliance and enforcement activities regarding drugs. Links to pages you that may find useful are provided below.

I look forward to consulting with you and other stakeholders as we continue to make more regulatory information publicly available.

Sincerely,

Deryck Trehearne
Director General
Resource Management and Operations Directorate
Health Products and Food Branch
Health Canada

Weblinks:

Drugs and Health Products
http://healthycanadians.gc.ca/drugs-products-medicaments-produits/index-eng.php

Drug Product Database (includes access to Product Monographs)
http://webprod5.hc-sc.gc.ca/dpd-bdpp/index-eng.jsp

Canadian Vigilance Adverse Reaction Online Database
http://webprod3.hc-sc.gc.ca/arquery-rechercheei/index-eng.jsp
